# Extramedullary relapse of acute lymphoblastic leukemia treated with a CAR-T cell therapy bridge to unrelated cord blood transplantation: a case report and review of the literature

**DOI:** 10.3389/fonc.2025.1508676

**Published:** 2025-08-20

**Authors:** Huibo Li, Jie Liu, Dan Guo, Yanqiu Zhao, Qi Li, Sen Qi, Jinxiao Hou, Jianping Zhang, Shengjin Fan

**Affiliations:** ^1^ Division of Hematology, Department of Medicine, The First Affiliated Hospital of Harbin Medical University, Harbin, China; ^2^ Hematology Department, the Second Hospital of Anhui Medical University, Hefei, Anhui, China; ^3^ Department of Bone Marrow Transplantation, HeBei Yanda Lu Daopei Hospital, Langfang, China; ^4^ NHC Key Laboratory of Cell Transplantation, The First Affiliated Hospital, Harbin Medical University, Harbin, China

**Keywords:** extramedullary relapse, acute lymphoblastic leukemia, chimeric antigen receptor T cell, unrelated cord blood transplantation, PET/CT

## Abstract

Extramedullary relapse of acute lymphoblastic leukemia (ALL) is usually associated with poor prognosis. Chimeric antigen receptor T cell (CAR-T cell) therapy followed by allogeneic hematopoietic stem cell transplantation is beneficial for relapsed/refractory (r/r) B cell acute lymphoblastic leukemia (B-ALL). Here, we report a B-ALL patient with extramedullary relapse involving several organs, including multiple lymph nodes and the breast, kidney, uterus and pancreas. After treatment with CAR-T cell therapy, positron emission tomography/computed tomography (PET/CT) revealed that she went into remission, with an almost undetectable tumor mass. She subsequently received unrelated cord blood transplantation (UCBT). Although she achieved minimal residual disease (MRD)-negative remission after UCBT for 5 months, she relapsed at the 6th month after UCBT. This patient achieved remission after subsequent interferon-α treatment for two weeks but eventually died of severe pneumonia. This case highlights the possibility of unusual relapse sites after chemotherapy and that regular biopsy of the mass is not sufficient to assess the scope and location of recurrence. PET/CT may be a useful tool to monitor the scope of extramedullary recurrence and follow-up remission. Further understanding of the pathology of extramedullary relapse is warranted to improve the management of such challenging presentations. This case suggests the efficacy of CAR-T cell therapy combined with UCBT in adult B-ALL patients with extramedullary relapse.

## Introduction

Refractory/relapsed (r/r) B cell acute lymphoblastic leukemia (B-ALL) in adults has a dismal prognosis, with fewer than 10% of patients being long-term survivors (1). Although relapse is detected mainly in the bone marrow, extramedullary tissues are occasionally involved, accounting for 15~20% of all relapses (2). Commonly, extramedullary relapses (EMRs) involve the central nervous system (CNS) or testes. Unusual EMRs in ALL are relatively rare, and the outcomes of EMRs are poor because there are no accepted standard therapeutic approaches.

CD19-targeted chimeric antigen receptor T cell (CAR-T cell) therapy is a promising treatment for r/r B-ALL, including extramedullary relapse (3). Nevertheless, the long-term efficacy of CAR-T cell therapy remains unsatisfactory because of the high recurrence rate (4, 5). Allogeneic hematopoietic stem cell transplantation (allo-HSCT) is considered the only curative option for patients with r/r B-ALL after effective salvage therapy without measurable residual disease (MRD) (6).

Umbilical cord blood (UCB) has gradually been considered an alternative source of peripheral blood progenitor cells (PBPCs) or bone marrow (BM) transplantation, particularly when a human leukocyte antigen (HLA)-matched donor is not available. Previous studies have shown that umbilical cord blood transplantation (UCBT) is an option for improving the prognosis of patients who have undergone CAR-T cell therapy (7, 8). However, reports of CAR-T cell therapy followed by UCBT for the treatment of EMR involving multiple organs in r/r B-ALL patients are rare. Here, we describe a r/r B-ALL patient with EMR involving multiple organs who achieved remission after treatment with CAR-T cell therapy combined with UCBT but who relapsed with MRD positivity and eventually died of severe pneumonia.

## Case description

A 39-year-old female with fatigue was admitted to our hospital. Her peripheral blood revealed normocytic anemia (Hb 65 g/dL). Bone marrow (BM) aspiration revealed hypercellular marrow with 75% lymphoblastic leukemia cells negative for α-nonspecific esterase but positive for periodic acid-Schiff (PAS). Flow cytometric (FCM) immunophenotyping revealed that the leukemic blasts were positive for CD19, CD34, CD38, and TdT, with partial expression of HLA-DR, CD10, and CD22 but negative expression of cIgM (**Supplementary Figure S1A**). Cytogenetic evaluation revealed 47, XX, +8, del(9)(p22) in 20/20 metaphases. RNA sequencing revealed mutations in KRAS, ASXL2, TTN, KMT2D, and ASXL1 (**Supplementary Figure S1B**, **Supplementary Table S1**). The diagnosis of common B-ALL was determined, and the patient was subsequently treated with induction chemotherapy for the VICP regimen (4 mg of vindesine on Days 1, 8, 15 and 22; 10 mg of idarubicin on Days 1-3; 750 mg/m^2^ cyclophosphamide on Days 1 and 15; and 60 mg of prednisone on Days 1-28). After completion of the VICP regimen, she achieved MRD-negative complete remission (CR), and the test for central nervous system infiltration by FCM was negative. Meningeal prophylaxis was performed via intrathecal methotrexate and cytarabine administration. The patient was given 2 consolidation cycles of the Hyper CVAD A regimen (hyperfractionated cyclophosphamide, vindesine, doxorubicin, and dexamethasone) and 3 consolidation cycles of high-dose methotrexate with cytarabine treatment. An overview of further development is provided in **Supplementary Figure S2**.

**Figure 1:**
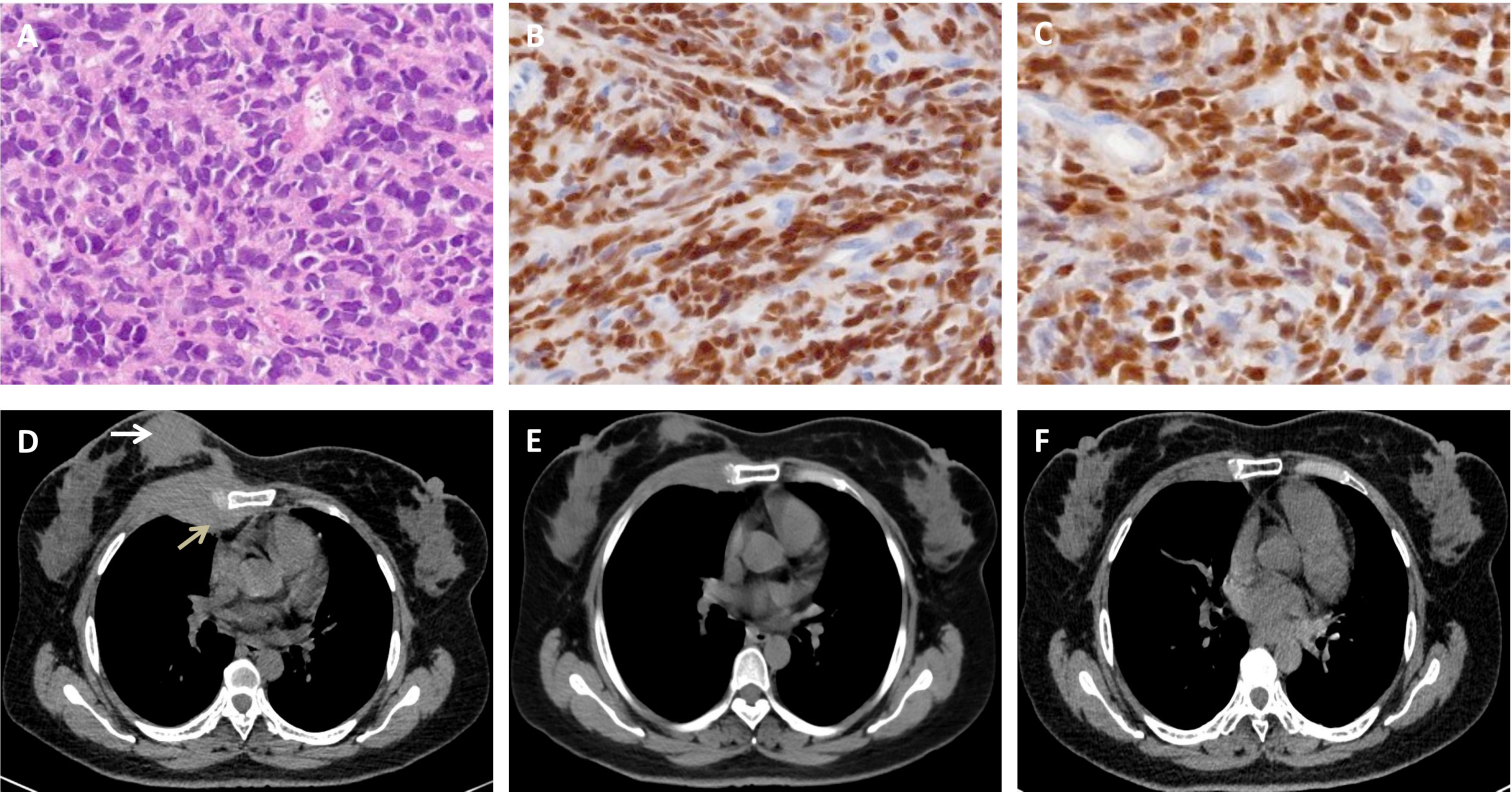
Biopsy of the breast mass and chest CT scan. **(A-C)**. Hematoxylin and eosin staining **(A)** and immunohistochemical staining of PAX-5 **(B)** and TdT **(C)** show typical lymphoblasts. **(D)**. Chest CT image showing that the breast mass (white arrow) extended into the right lung (gray arrow). **(E)**. The mass decreased after chemotherapy and radiotherapy. **(F)**. A chest CT scan revealed that the abnormal breast mass was significantly alleviated after CAR-T cell therapy.

**Figure 2:**
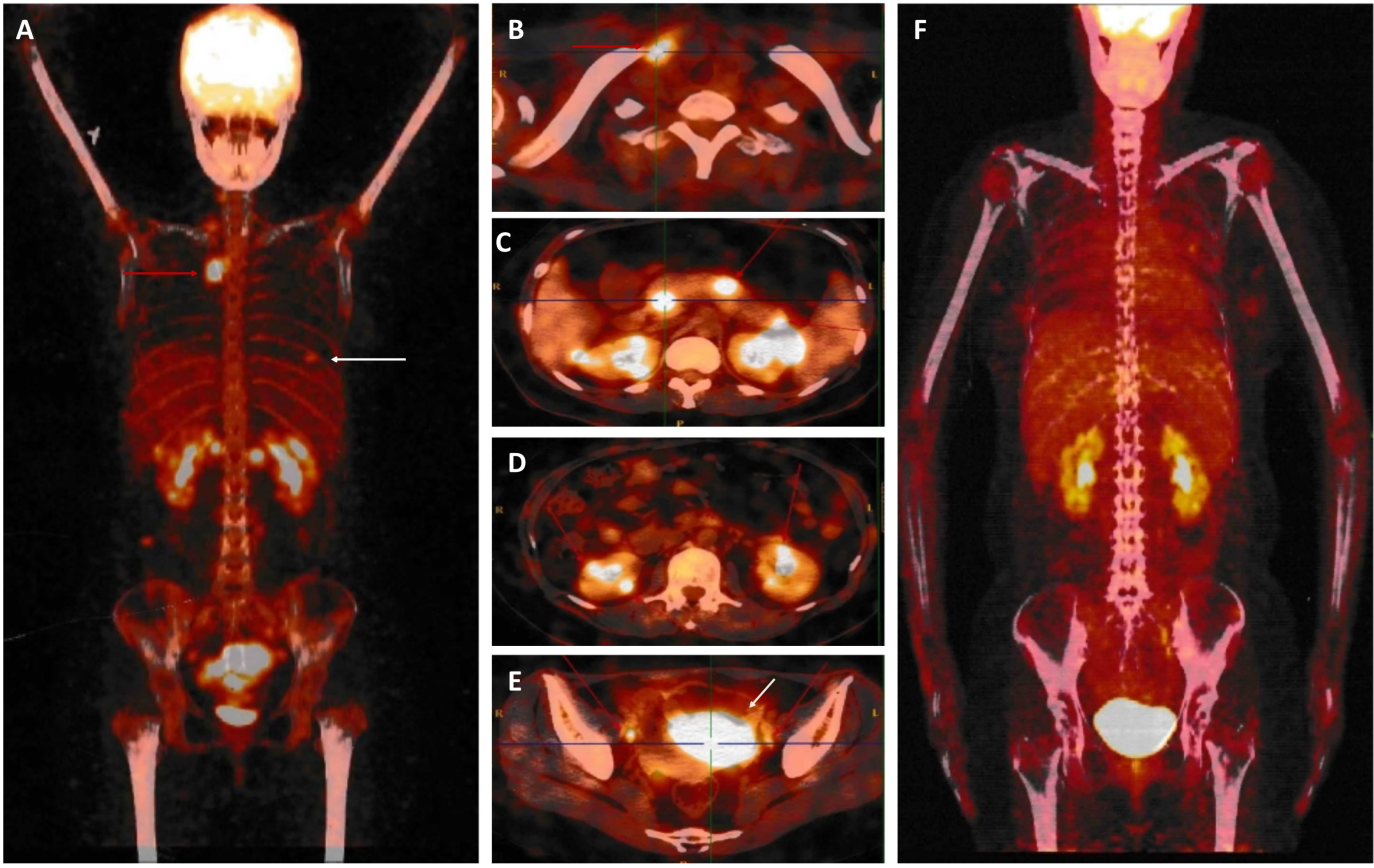
Whole body 18-fluorodeoxyglucose positron emission tomography-computed tomography. **(A-E)**. Before starting treatment with CAR-T cell therapy, PET/CT data revealed abnormally intense high-metabolic masses in the breast [white arrow in **(A)**], lymph nodes [red arrow in **(A, B, E)**], pancreas **(C)**, kidney **(D)**, and uterus [white arrow in **(E)**]. **(F)**. After CAR-T cell therapy, complete remission occurred.

After providing written informed consent, the patient was given a daily subcutaneous dose of GCSF (10 μg/kg/day) for 5 days and plerixafor (0.24 mg/kg) on the evening of Day 5 to mobilize autologous hematopoietic stem cell. The patient failed to achieve an adequate CD34^+^ cell count. After the second mobilization with GCSF and plerixafor, abnormal leukemic blast cells were detected in the peripheral blood stem cell apheresis product (0.01%). Abnormal leukemic blast cells were subsequently found via bone marrow aspiration (0.3%). After treatment with blinatumomab infusion for 28 days, the percentage of leukemic cells decreased to 0.02%. Selinexor (40 mg/week) and chidamide (60 mg/week) were subsequently administered for one month. At the same time, a right breast mass was found, which was revealed by biopsy to be extramedullary relapse (**Figures 1A–C**). Chest CT revealed that the mass extended from the right breast into the right lung (**Figure 1D**). The patient was subsequently treated with radiotherapy and chemotherapy, including dexamethasone, liposomal mitoxantrone, etoposide, and cyclophosphamide. After treatment, the right breast mass decreased (**Figure 1E**), and the patient was referred to our department for allogeneic HSCT. Unfortunately, positron emission tomography (PET)-CT images before starting the conditioning regimen revealed extensive extramedullary relapse involving multiple lymph nodes, the breast, kidney, uterus, and pancreas. Peripheral blood lymphocytes were then collected from the patient to prepare anti-CD19 CAR-T cells at HeBei Yanda Lu Daopei Hospital. After pretreatment with the FC regimen (30 mg/m^2^ fludarabine daily for 3 days and 300 mg/m^2^ cyclophosphamide daily for 3 days), anti-CD19 CAR-T cells were infused at a total dose of 1.0x10^7^/kg for 3 consecutive days as previously reported (9). One month after CAR-T cell therapy, the patient achieved complete remission without MRD in the bone marrow, and a PET–CT scan suggested complete resolution of extramedullary disease (**Figure 2**). CT scan also showed the regression of right breast mass (**Figure 1F**). An overview of MRD detected by FCM is shown in **Supplementary Figure S3**.

**Figure 3:**
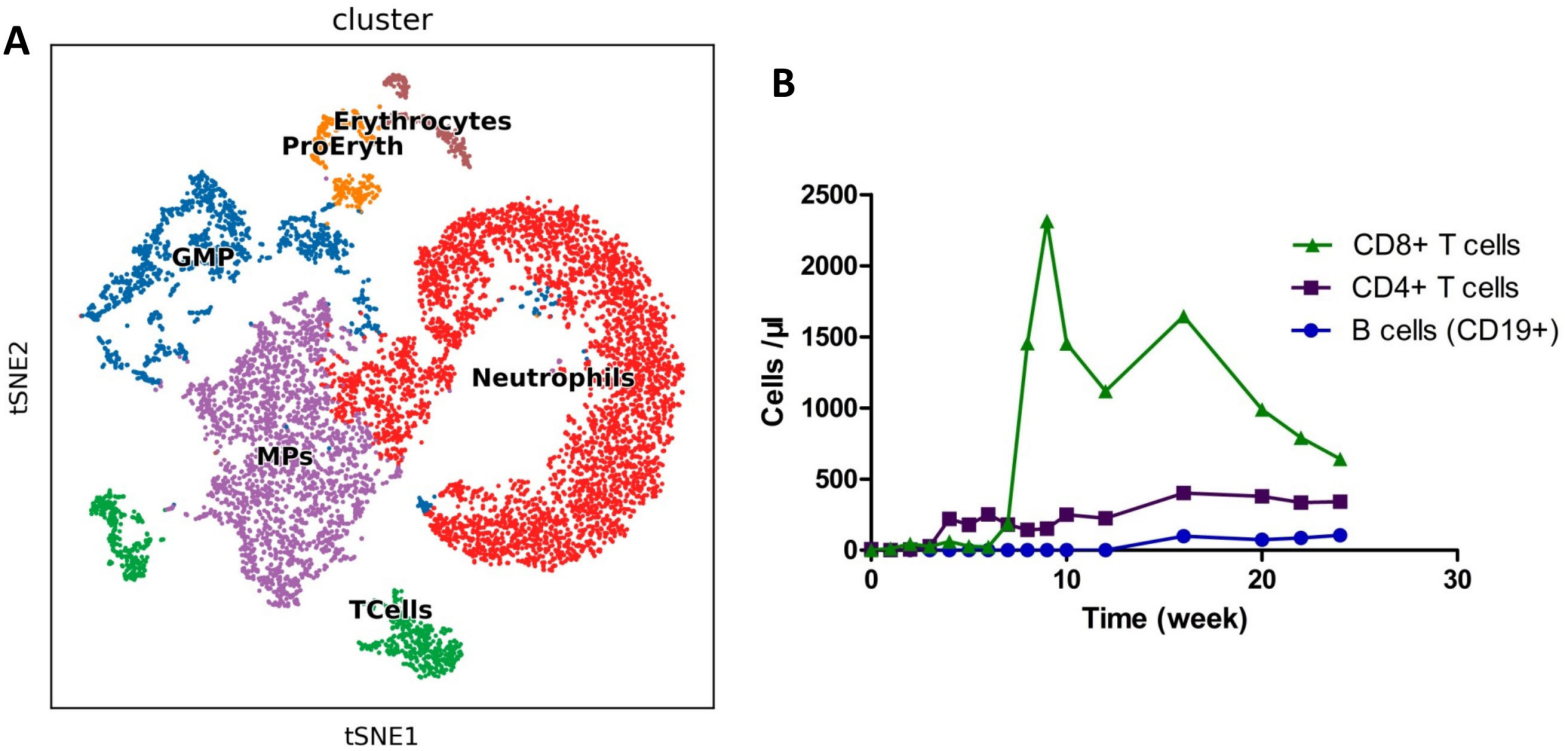
Detection of immune cells after allo-HSCT. **(A)**. Landscape of bone marrow cell subpopulations by single-cell sequencing on Day 28. **(B)**. Circulating immune cells were measured by flow cytometry as absolute numbers after transplantation. The normal ranges of B cells, CD4+ T cells, and CD8+ T cells were 95~106, 404~1612, and 220~1129 cells/μl, respectively.

The patient subsequently underwent an UCBT at our department. Allografts were from 8/10 HLA-matched unrelated umbilical cord blood. The doses of total nucleated cells and CD34+ cells infused were 1.06x10^7^/kg and 6.0x10^5^/kg, respectively. The conditioning regimen included 5 mg/kg thiotepa for 1 day, 5 mg/m^2^ cladribine daily for 4 days, intravenous 3.2 mg/kg/d busulfan for 4 days, and intravenous 50 mg/kg melphalan daily for 2 days. Cyclosporine and mycophenolate mofetil were used for GVHD prophylaxis. Neutrophil and platelet engraftment was observed on Days 19 and 90 after umbilical cord blood infusion, respectively. On Day 28, after UBCT, the patient was MRD negative and had complete donor chimerism. B cell aplasia was detected via single-cell sequencing (Singleron Biotechnologies) as described previously (10) on Day 28 and persisted for 5 months after UCBT (**Figure 3**). The bilirubin and transaminase levels of the patient increased on Day 21 and decreased to normal levels following treatment with systemic glucocorticoids. The patient developed cytomegalovirus (CMV) viremia on Day 53, and the serum CMV-DNA test became negative two weeks later. The patient remained MRD negative with complete donor chimerism for 5 months after UCBT. At the 6th month, abnormal B cells were detected (0.05%) in 2 consecutive bone marrow samples within a 2-week interval. No evidence of extramedullary recurrence was observed. The patient was subsequently given 3 million IU of interferon-α three times a week, as previous reported (11). She achieved MRD negativity again after 14 days of interferon-α treatment. The FCM results for the bone marrow are shown in **Supplementary Figure S3**. Unfortunately, the patient was transferred to the intensive care unit because of severe pneumonia (**Supplementary Figure S4**) and subsequently died.

## Discussion

This case report discusses the use of CAR-T cell therapy for bridging UCBT in an r/r B-ALL patient with extramedullary relapse in multiple organs. The most common locations of extramedullary relapse are the CNS and testes; other locations of extramedullary relapse, such as the breast, kidney, uterus and pancreas, are rare (12, 13). It is unknown whether the lesion in the patient was present before blinatumomab treatment, as routine PET–CT imaging is not the standard of care. The incidence of extramedullary relapse is reportedly greater following blinatumomab treatment (14). PET imaging for detecting extramedullary disease (EMD) in acute leukemia patients is sensitive but not specific due to false positives resulting from inflammation or infection (15). The use of PET imaging has been reported in a small number of patients for both the diagnosis and treatment response of EMD, but further study is needed (16, 17). Thus far, there are no standard management protocols for patients with extramedullary relapse that may improve dismal outcomes. Even with intensive chemotherapy, radiation therapy, and HSCT, a cure remains challenging (18).

There are reports of ALL patients with non-CNS extramedullary relapse who have been successfully treated with CAR-T cell therapy, including patients with testicular, skin, renal, pancreatic, pleural, breast, and bony diseases (19–21). Additional clinical trials using CAR-T cell therapy for patients with combined and isolated extramedullary relapsed ALL have been performed (22–24). Here, we reported that an r/r B-ALL patient with EMR in multiple organs achieved significant remission after treatment with CAR-T cell therapy.

While CAR-T cell therapy increases the remission rate of patients diagnosed with r/r B-ALL, the limited duration of remission and unsatisfactory long-term survival present challenges for CAR-T cell therapy. Several studies have shown that consolidation with HSCT after CAR-T cell therapy can improve leukemia-free survival (LFS), improve event-free survival (EFS), and decrease the relapse rate (25, 26). Some studies demonstrated that consolidative allo-HSCT has better overall survival (OS) than CAR-T cell therapy alone (26–28), while others suggest that the potential advantages might be offset by the risks of transplant-associated non-relapse mortality. A recent study with long-term follow-up revealed the long-term efficacy of allo-HSCT as consolidation therapy post CAR-T (29). Umbilical cord blood is gradually being considered an alternative source of peripheral blood progenitor cells for bone marrow transplantation, particularly when a human leukocyte antigen (HLA)-matched donor is not available (7). Recently, studies have shown that CAR-T cell therapy combined with UCBT can improve the clinical outcomes of patients with r/r B-ALL (7, 8). The time from CAR-T cell therapy to transplantation is associated with the risk of death (30). Some researchers have recommended treatment with consolidative allo-HSCT within three months of CAR-T cell therapy to maximize the benefit (30, 31). The present patient was treated with consolidative UCBT within three months of CAR-T cell therapy. However, a study on UCBT as a consolidative treatment for CAR-T cell therapy has revealed no correlation between the interval and long-term survival (8), but the number of patients included in this previous study was small (8). The optimal time for consolidative UCBT needs to be determined by multicenter and large-sample studies. Based on the present case, consolidative UCBT after CAR-T cell therapy may be considered an option to improve the prognosis of r/r B-ALL patients with EMR involving multiple organs.

Lower B cell counts have been reported to be associated with higher risks of death and relapse (32). Low B cell counts on Day 80 are associated with high infection rates between Days 100 and 365 after allogeneic marrow transplantation (33). B cell aplasia is not found in CAR-T cells serves as a reduced-intensity conditioning regimen for haploidentical stem cell transplantation (34). However, the number of B cells in the present case was lower than normal until 5 months after UBCT. The reason for delayed B cell reconstitution may be acute GVHD and cytomegalovirus infection, as reported previously (26). This may be one of the reasons for the severe pneumonia in our patient, indicating the need for more sensitive laboratory tests to detect the underlying causes of pneumonia.

In conclusion, extramedullary relapse in acute lymphoblastic leukemia patients is uncommon, with the involvement of multiple organs being extremely rare. Although PET-CT is not generally used for ALL, it can be useful in identifying unusual EMR. For EMR in r/r B-ALL, CAR-T cell therapy may be a good salvage treatment, and UCBT may also be an option for consolidation. However, further research is needed to improve the prognosis of EMR involving multiple organs in adults with B-ALL.

## Data Availability

The original contributions presented in the study are included in the article/**Supplementary Material**. Further inquiries can be directed to the corresponding author.

## Supplementary material

Supplementary Figure 1 Flow cytometry and G-banding analysis of the patient. **(A)**. Flow cytometry results of the marrow at the first visit. **(B)**. G-banded karyotype showing chromosome aberrations (arrows).

Supplementary Figure 2 Timeline of the patient’s history. BiTE, blinatumomab; Ser, selinexor; Chi, chidamide; CART-T cell, chimeric antigen receptor T cell; MRD, minimal residual disease; UCBT, umbilical cord blood transplantation.

Supplementary Figure 3 Minimal residual disease (MRD) of the bone marrow aspirate was detected via flow cytometry after mobilization. BiTE, blinatumomab; UCBT, umbilical cord blood transplantation.

Supplementary Figure 4 Lung CT scan of the patient.
