# Supplementary figures and images for: Extramedullary relapse of acute lymphoblastic leukemia treated with a CAR-T cell therapy bridge to unrelated cord blood transplantation: a case report and review of the literature
Source: Front Oncol. 2025 Aug 20;15:1508676. doi: 10.3389/fonc.2025.1508676 (PMC12405284; doi:10.3389/fonc.2025.1508676)

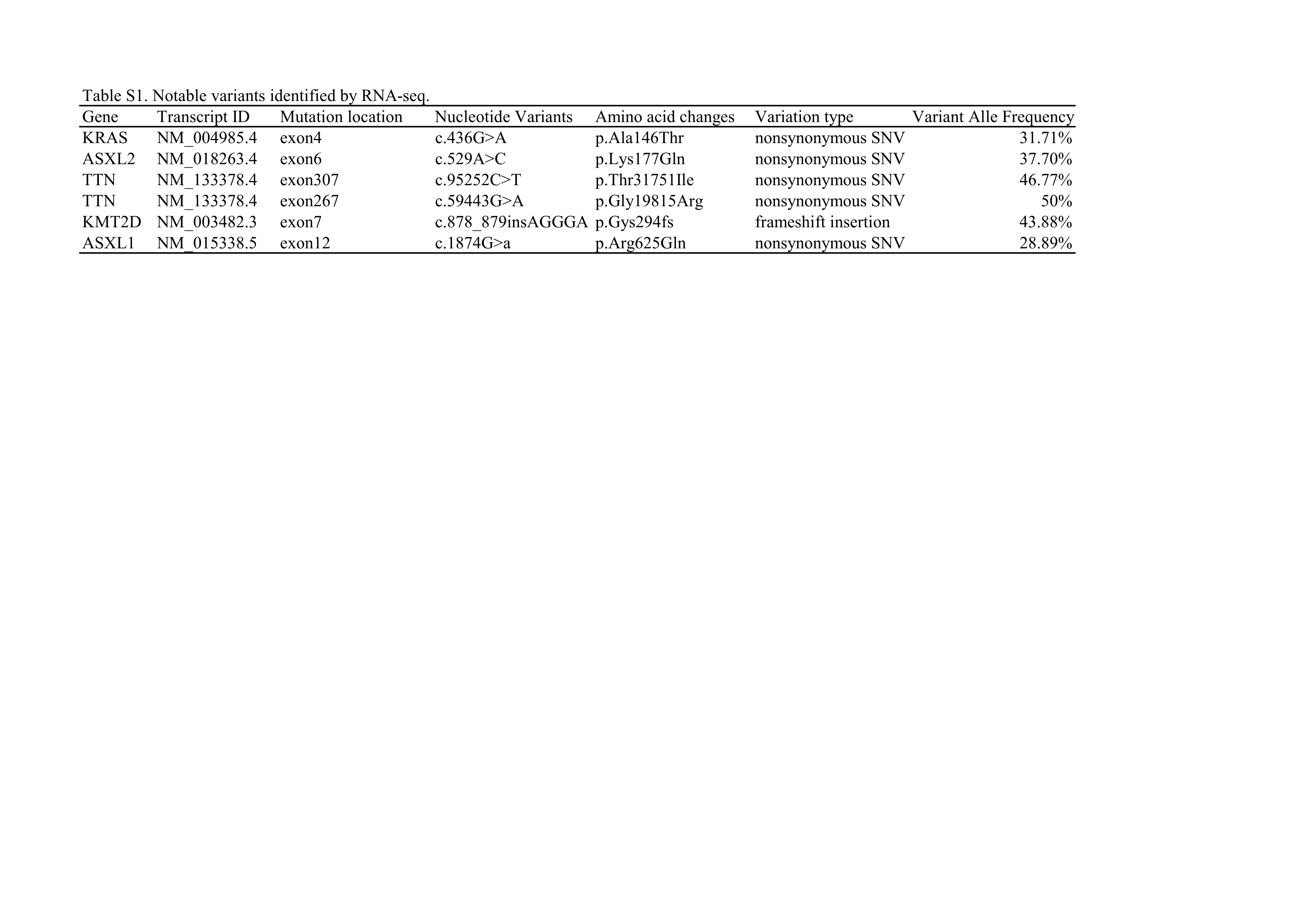

Supplement: Supplementary Figure 1 — Flow cytometry and G-banding analysis of the patient. (A). Flow cytometry results of the marrow at the first visit. (B). G-banded karyotype showing chromosome aberrations (arrows). [file Image1.jpeg]

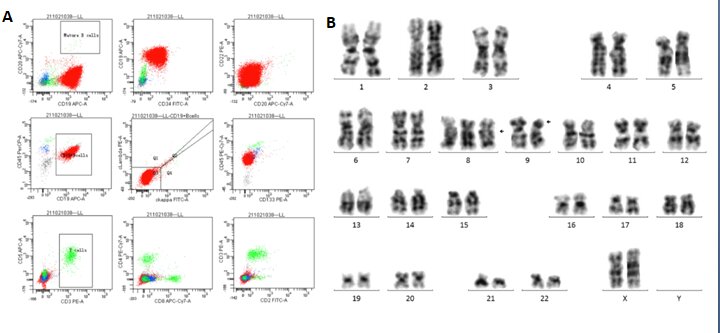

Supplement: Supplementary Figure 2 — Timeline of the patient’s history. BiTE, blinatumomab; Ser, selinexor; Chi, chidamide; CART-T cell, chimeric antigen receptor T cell; MRD, minimal residual disease; UCBT, umbilical cord blood transplantation. [file Image2.jpeg]

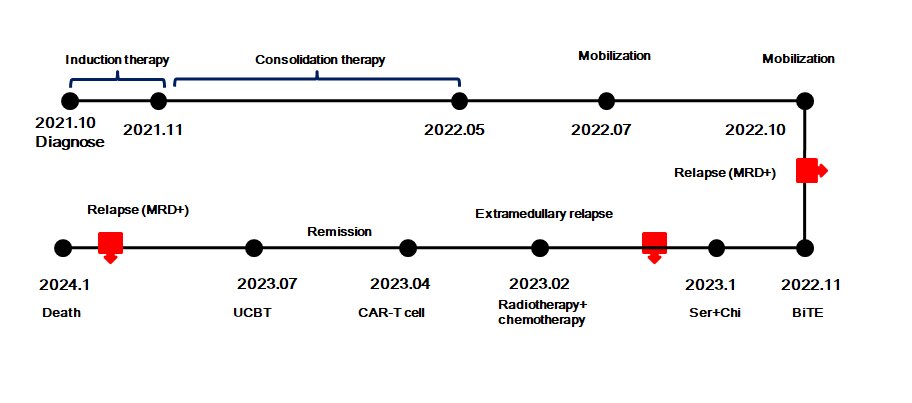

Supplement: Supplementary Figure 3 — Minimal residual disease (MRD) of the bone marrow aspirate was detected via flow cytometry after mobilization. BiTE, blinatumomab; UCBT, umbilical cord blood transplantation. [file Image3.jpeg]

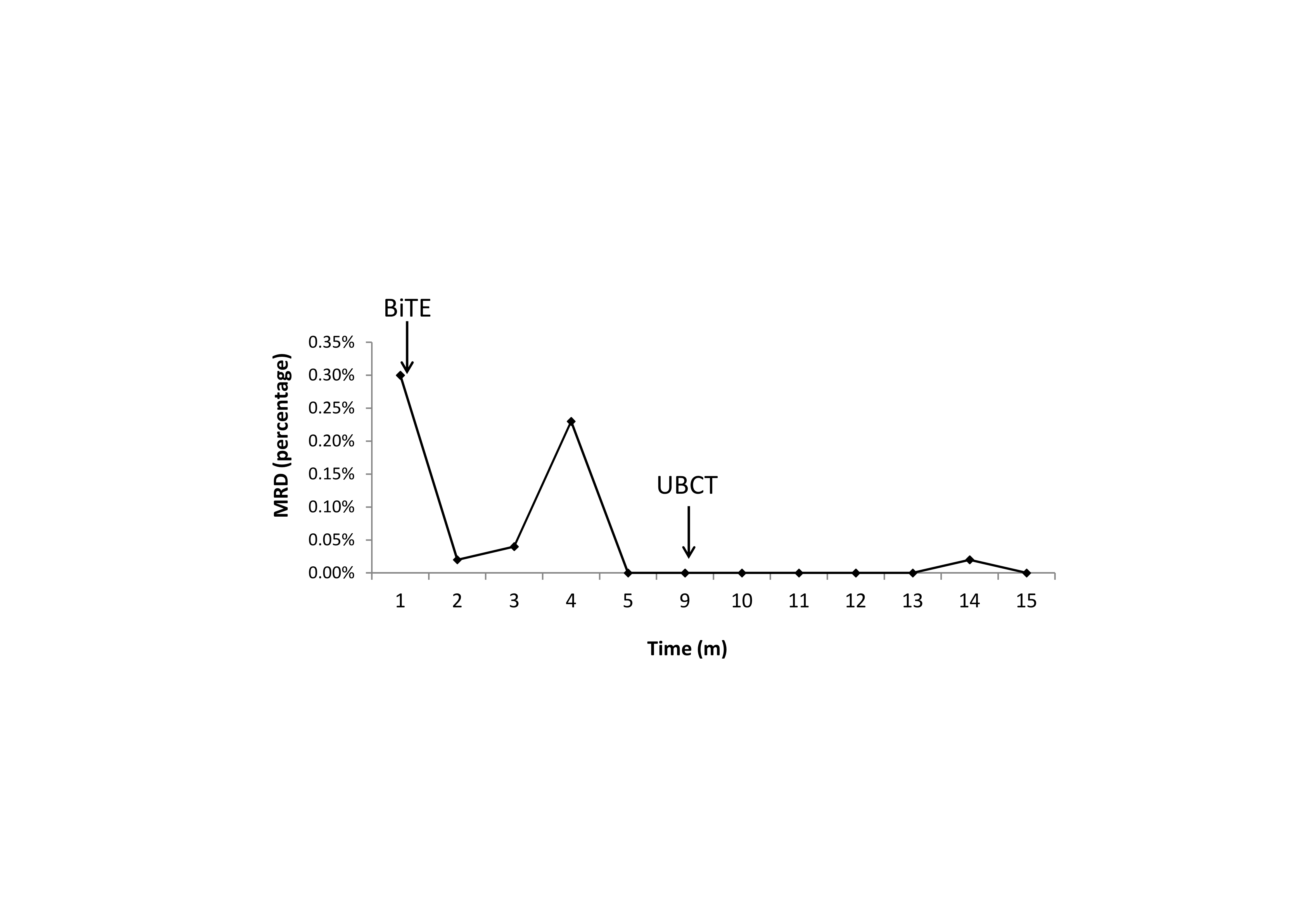

Supplement: Supplementary Figure 4 — Lung CT scan of the patient. [file Image4.jpeg]

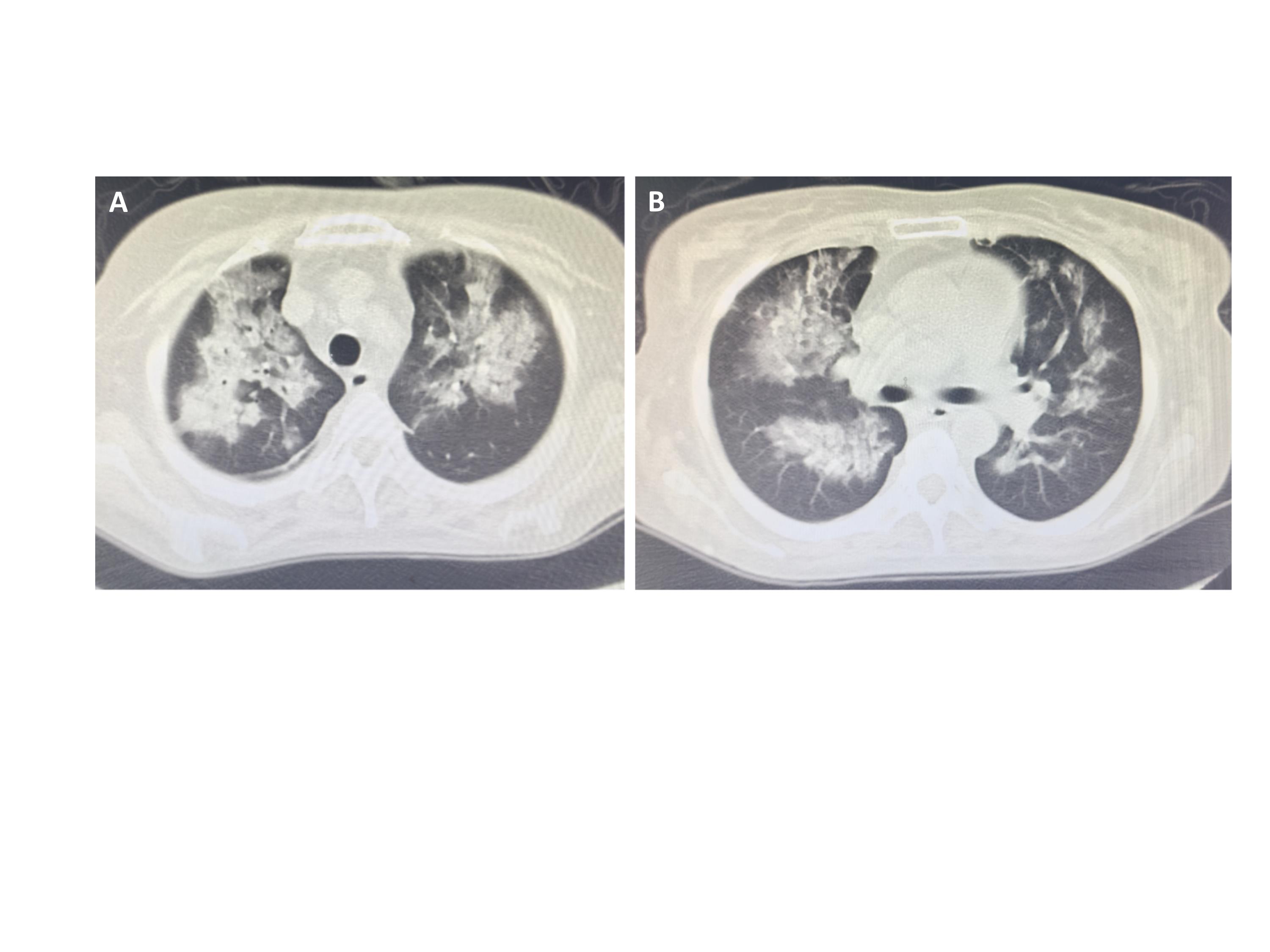

Supplement: Supplementary file 5 [file Image5.jpeg]
